# Identifying actionable strategies: using Consolidated Framework for Implementation Research (CFIR)-informed interviews to evaluate the implementation of a multilevel intervention to improve colorectal cancer screening

**DOI:** 10.1186/s43058-021-00150-9

**Published:** 2021-05-31

**Authors:** Helen Lam, Michael Quinn, Toni Cipriano-Steffens, Manasi Jayaprakash, Emily Koebnick, Fornessa Randal, David Liebovitz, Blasé Polite, Karen Kim

**Affiliations:** 1 Center for Asian Health Equity, University of Chicago, 5841 S Maryland Ave, Rm S406, MC 1140, Chicago, IL 60637 USA; 2 Department of Internal Medicine Section of General Internal Medicine, University of Chicago, 5841 S Maryland Ave, Chicago, IL 60637 USA; 3 University of Chicago Medicine, 5841 S. Maryland Ave, MC 2115, Suite G109, Chicago, IL 60637 USA; 4 Division of General Internal Medicine & Geriatrics, Feinberg School of Medicine, Northwestern University, 750 N. Lake Shore Dr., 10th Floor, Chicago, IL 60611 USA; 5 University of Chicago Medicine Hematology and Oncology, 5841 S Maryland Ave, Chicago, IL 60637 USA

**Keywords:** Federally Qualified Health Center, Implementation, Colorectal cancer screening, Evidence-based intervention, Consolidated Framework for Implementation Research, Implementation strategy

## Abstract

**Background:**

Many evidence-based interventions (EBIs) found to be effective in research studies often fail to translate into meaningful patient outcomes in practice. The purpose of this study was to identify facilitators and barriers that affect the implementation of three EBIs to improve colorectal cancer (CRC) screening in an urban federally qualified health center (FQHC) and offer actionable recommendations to improve future implementation efforts.

**Methods:**

We conducted 16 semi-structured interviews guided by the Consolidation Framework for Implementation Research (CFIR) to describe diverse stakeholders’ implementation experience. The interviews were conducted in the participant’s clinic, audio-taped, and professionally transcribed for analysis.

**Results:**

We used the five CFIR domains and 39 constructs and subconstructs as a coding template to conduct a template analysis. Based on experiences with the implementation of three EBIs, stakeholders described barriers and facilitators related to the intervention characteristics, outer setting, and inner setting. Implementation barriers included (1) perceived burden and provider fatigue with EHR (Electronic Health Record) provider reminders, (2) unreliable and ineffectual EHR provider reminders, (3) challenges to providing health care services to diverse patient populations, (4) lack of awareness about CRC screening among patients, (5) absence of CRC screening goals, (6) poor communication on goals and performance, and (7) absence of printed materials for frontline implementers to educate patients. Implementation facilitators included (1) quarterly provider assessment and feedback reports provided real-time data to motivate change, (2) integration with workflow processes, (3) pressure from funding requirement to report quality measures, (4) peer pressure to achieve high performance, and (5) a culture of teamwork and patient-centered mentality.

**Conclusions:**

The CFIR can be used to conduct a post-implementation formative evaluation to identify barriers and facilitators that influenced the implementation. Furthermore, the CFIR can provide a template to organize research data and synthesize findings. With its clear terminology and meta-theoretical framework, the CFIR has the potential to promote knowledge-building for implementation. By identifying the contextual determinants, we can then determine implementation strategies to facilitate adoption and move EBIs to daily practice.

Contributions to the literature

- This research study identified and described implementation barriers and facilitators specific to implementing evidence-based intervention at a large urban federally qualified health center. The barriers we identified in this study are common in clinical practices. We recommended implementation strategies to mitigate these barriers.
- We found that facilitators that were unique to federally qualified health centers, including the requirement from funders to submit quality measures, the incentive for improvement, and the providers’ commitment to their patients, and all could promote changes and openness to new ways of practice as well as have the potential to mitigate resistance and accelerate the implementation process.
- The Consolidated Framework for Implementation Research (CFIR) is well suited to evaluate the implementation process of evidence-based interventions (EBIs) to improve a cancer screening program based in an urban federally qualified health center. Data were coded to CFIR constructs without requiring any adaptation, and barriers were easily identified. Thus, CFIR serves well as a pragmatic guiding framework for evaluation.

## Background

Gaps still exist between identifying evidence-based intervention (EBIs), changes to practice, and improving outcomes for patients [1, 2]. EBIs for promoting cancer screening include patient-, provider-, and organization-oriented approaches are no exception. Many of these EBIs found to be effective in research studies often fail to translate into meaningful patient outcomes in practice due to the difficulty of translating EBIs into the daily clinical workflow [3]. This failure is particularly evident among safety-net health systems, such as federally qualified health centers (FQHCs) that provide care to low-income, uninsured, and minority patients due to resource constraints, lack of support, and competing demands. The implementation of EBIs is a complex process [4]. It involves attention to various factors at different levels related to the intervention itself, the local implementation context, interactions within and across health care delivery organizations, and the strategies used to implement the interventions [5–7]. Implementation strategies used to implement an intervention are the “how-to” component of changing healthcare practice [8]. Studying the implementation process can yield critical information on the determinants that influence implementation and, subsequently, the outcome achievement [9–11]. However, large knowledge gaps remain regarding “how-to” move EBIs into daily practice.

Numerous theories and models have been proposed to assess potential contextual determinants and inform the implementation of innovations [12, 13]. The Consolidated Framework for Implementation Research (CFIR) [14] is a well-operationalized and widely used framework to assess potential barriers and facilitators within local settings. This study aimed to (1) use CFIR to identify facilitators and barriers affecting the implementation of three EBIs with a large urban FQHC, (2) offer actionable implementation strategies to improve the EBI’s implementation efforts in a new study, and (3) expand the implementation science literature regarding the feasibility of using CFIR as a pragmatic guiding framework for an evaluation and a template to organize research data. Two of the EBIs were “provider-oriented,” meaning they increased the likelihood that providers would recommend screening; these EBIs were provider reminders and provider assessments and feedback [15, 16]. However, completion of screening involves patient compliance with provider recommendations. The third EBI was patient navigation (CRC steward), which has been widely used to improve CRC screening compliance [17–25] and recommended by NIH as an evidence-based strategy for CRC screening [26]. By interviewing diverse stakeholders across four primary care clinics, we aimed to describe factors that hinder or promote the implementation of EBIs in order to improve the rates of CRC screening.

## Methods

This study is part of a larger program entitled “Accelerating Colorectal Cancer Screening and Follow-up through Implementation Science-Chicago (ACCSIS-Chicago).” The ACCSIS-Chicago project is part of the NCI-funded consortium, the Accelerating CRC Screening and Follow-up through Implementation Science (ACCSIS) Program. The overall aim of ACCSIS is to conduct multi-site, coordinated, and transdisciplinary research to evaluate and improve CRC screening processes using implementation science strategies. The ACCSIS-Chicago Program aims to implement a multilevel, multicomponent intervention to increase rates of CRC screening, follow-up, and referral-to-care at four FQHCs located in Illinois and Indiana. Findings from this study are used to inform the implementation process of the multilevel intervention in these four FQHCs. This study has been reviewed and approved by the University of Chicago Institutional Review Board (IRB18-1141).

### Conceptual framework

The Consolidated Framework for Implementation Research (CFIR) synthesized and categorized constructs across different theories and models and provided a meta-theoretical framework to advance our understanding of implementation across various settings and interventions. The CFIR is composed of five major domains: (1) intervention characteristics, (2) inner setting, (3) outer setting, (4) characteristics of individual involved in the implementation, and (5) implementation process. There are 39 CFIR constructs and subconstructs under these five domains, which reflect the evidence base of factors most likely to affect the implementation of interventions [14]. Much of the research using the CFIR to date has been qualitative [27–34], with some studies using the CFIR to organize emerging themes following data collection [35–37]. The CFIR can also be used to guide formative evaluations and exploration into the question of which factors influenced implementation and how implementation influenced the performance of the intervention.

### Study design

**Fig. 1:**
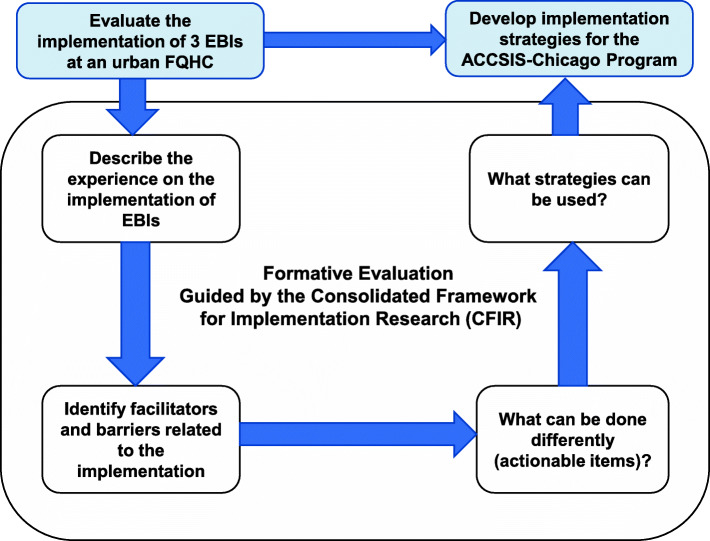
Conceptual framework for the study

In this study, we conducted a formative evaluation using a qualitative study design to gain insights into the implementation process. Clinic providers and staff members involved in the implementation process were selected for one-on-one semi-structured interviews. We used CFIR to guide the evaluation process, from developing interview questions and organizing the coding tree to analyzing data and summarizing findings [14]. Through qualitative interviews with diverse stakeholders, we aimed to describe the implementation experience, identify factors that hindered or facilitated the implementation process, and offer mitigation strategies to improve the implementation process. Figure 1 shows the conceptual framework for this qualitative study.

### Study setting

We conducted semi-structured interviews to assess the implementation experiences of three EBIs that were ongoing in four primary care clinics in an urban FQHC. The FQHC has 11 clinics, with 8 providing primary care services. In 2018, the FQHC provided care to 26,102 unique patients. Over 82% of patients came from racial/ethnic minority groups, nearly one third of patients were uninsured (32%), and 32% were best served in a language other than English. At the beginning of 2016, the FQHC participated in a Centers for Disease Control and Prevention (CDC) funded CRC Control Program and started implementing three EBIs to improve screening rates at its primary care clinics with technical support from the University of Chicago program team. The three EBIs implemented to promote CRC screening include (1) provider reminder, which was generated manually by staff members along with an automated reminder from the Electronic Health Record (EHR); (2) quarterly provider assessment and feedback with the clinic- and individual provider-specific reports capturing CRC screening order rates and screening completion rates; and (3) CRC stewards (integrated care specialists) to identify patients who needed CRC screening, provide patient education and follow-up with patients to ensure compliance with screening orders. CRC screening rate at the FQHC has been improving, changing from 33.6% in 2016 and 37% in 2017 to 41% by the end of 2018.

### Study sample

The FQHC had a total of 48 primary care providers in September 2018 when the sampling procedure began. The primary care providers included 22 physicians, 20 advanced nursing practitioners, and 6 physician assistants. We selected 8 physicians, 4 advanced nursing practitioners, and 2 physician assistants based on their overall individual CRC screening order rates in 2017. Of the 14 providers, 6 were high performers with an order rate at the top 25%, and 4 were low performers with an order rate at the lowest 25%. The range of individual CRC order rates from the selected primary care providers was between 25% and 81%, and the average order rate was 41%. The sample also included all four CRC stewards (integrated care specialists) and two administrators. We requested one on one interviews with primary care providers, CRC stewards (integrated care specialists), and administrators via email. The semi-structured interviews were conducted between November 2018 and December 2018. Participants who completed the interview received an honorarium of $50 for their time and efforts. Of the 14 primary care providers invited for the interviews, three providers no longer worked at the FQHC at the time of interviews, and one refused to participate. Thus, the final sample of the semi-structured interviews included ten primary care providers, four CRC stewards (integrated care specialists), and two administrators.

### Data collection

We used the publicly available CFIR Interview Guide Tool to inform our semi-structured interview guide [38]. The semi-structured interview guide included questions within the five CFIR domains and items relevant to the study and the implemented EBIs (Table 1). Interviews began by describing the CRC Control Program and the three EBIs implemented in the interviewees’ clinics. Five trained qualitative researchers (HL, MQ, BP, TC, EK) conducted in-person, semi-structured interviews with key informants between November 2018 and December 2018. The semi-structured interviews lasted 25 min to 45 min and were audio-recorded and professionally transcribed. All interviews were conducted in the participant’s clinic. Data collection continued until all selected participants completed the interviews (Table 1).

**Table 1:** CFIR-guided semi-structured interview questions

CFIR domain	Semi-structured interview question
**Intervention characteristics**	• How does the CRC control program (implement 3 EBIs) compare with other existing programs in your clinic? ○ What advantages does this CRC control program have? ○ What disadvantages does this CRC Control Program have? • What kind of changes did you have to make to the EBIs so they would work in your clinic? • How complicated were the EBIs? • How well has the EBIs been received in your clinic?
**Outer setting**	• How well does the CRC control program meet the needs of your patients? In what ways? • In your clinic, has there been a strong need to increase CRC screening rate? Why or why not?
**Inner setting**	• What supports were available to help you to adopt the EBIs? • In your clinic, what kinds of incentives are there for making the implementation of EBIs successful? • To what extent does your clinic set goals for the CRC control program? • How do you think your clinic’s culture affects the implementation of these EBIs?
**Characteristics of individual**	• What has been your motivation for wanting to help ensure the implementation is successful? • How confident are you about being able to use the EHR reminder (one of the EBIs) regularly in your clinic? How confident are your colleagues?
**Process**	• How do the EBIs fit with the workflow in your clinic? • What kinds of information did you collect as you worked on improving your clinic’s CRC screening rate? How was that information used?

### Data analysis

**Fig. 2:**
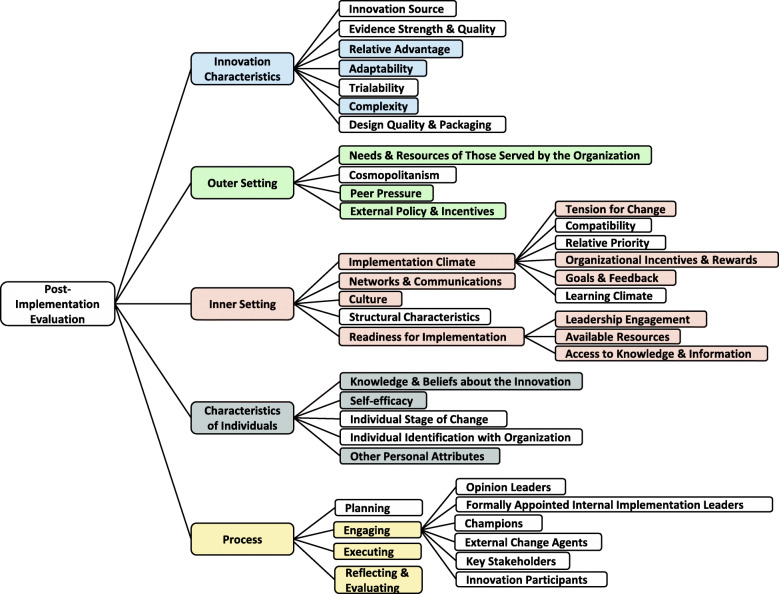
Coding tree based on the CFIR constructs

We conducted a template analysis of interview transcripts to identify themes describing facilitators and barriers to implementing the three EBIs related to CFIR constructs. Template analysis is a form of thematic analysis that emphasizes hierarchical coding and allows a relatively high degree of structure in analyzing the textual data with the flexibility to meet a particular study’s needs [39]. Instead of developing a coding template using a subset of data for the study, we adopted the CFIR constructs as our coding template. Figure 2 shows the coding tree for this qualitative study with codes that were identified from the data analysis. The 39 CFIR constructs and subconstructs were identified as a priori codes for an initial codebook. The colored boxes were codes identified in the interviews.

Before starting the coding process, the coders (HL, MQ, TC, EK) reviewed and discussed the CFIR coding definition, inclusion criteria, and exclusion criteria to come to a collective understanding of the codes. Due to the expansiveness of CFIR constructs, two coders independently double-coded the same transcripts and met regularly to review coding consistency and discuss problematic constructs. The coding team met together after all the transcripts were coded to discuss preliminary themes to reach a consensus. All coding and analysis were conducted in NVivo 12.

## Results

We conducted interviews with 16 stakeholders involved in various aspects of the implementation process, such as monitoring the implementation, championing the EBIs, and carrying out the EBIs. Table 2 summarizes our findings and identifies facilitators and barriers with quotations from our participants by the CFIR domains and constructs and the types of stakeholders (Table 2).

**Table 2:** Summary of findings by the Consolidated Framework for Implementation Research (CFIR) domains and constructs

	Innovation Characteristics	Outer Setting	Inner Setting	Characteristics of Individuals	Process
**Provider (MD, NP, PA) (*n*** **= 10)**	**1. Relative Advantage** Facilitator: *“When we get this data, right, you’re rejuvenated and you’re like, ‘Oh! We’ve really got to work on this.’ And, the hope is then it, we can incorporate it more and that it turns into more of a workflow type, I guess, moving forward that it fits back in workflow.”* Barrier: *“It (EMR reminder) didn’t rock anyone’s world either way. I don’t think. But it quickly fatigued people…I’d be like going into a chart at the end of the day, and I’d like, ‘Oh, yeah, I did see that,’ but I didn’t address it.”* *“I don’t know that it (EMR reminder) fit into our workflow specifically. I mean, we would see it popup. I don’t think it was overly effective…oftentimes we click through, we skim through that. But again, when the EMR reminders weren’t updated, even if the patient had the FIT test done, they become really unreliable and useless.”* **2. Adaptability** Facilitator: *“Provider feedback is usually done in the provider meeting. I think that we listen to people, we hear what’s going on, respond to it, try to come up with solutions. Frequently there is the leader or the EMR expert user who’s leading the discussion, but others within round table come up with their own – you know, who have confronted similar challenges – suggest workaround solutions.”* Barrier: *“Some of the other, some clinics I think are able to roll this out more easily than others, just based on their structures or their staffing or other things that can be more cumbersome in other places.”* **3. Complexity** Facilitator: *“So this one (EMR reminder), I think it’s fairly straightforward, in that we need to have this conversation and that the benefits are pretty clear. The patients get screened, and we know about their health. So, and there’s been, I would say a medium amount of reminders about it. So not too overwhelming, but also not, like, out of sight completely.”*	**1. External Policy & Incentives** *“We are an FQHC, so we are HRSA-funded. And part of HRSA baseline grant is based on quality measures.”* **2. Peer Pressure** Facilitator: *“The other thing is if you, we look at our UDS measures in different ways. But one of the ways we look at it is quartile where we rank nationally amongst everyone else. And we’re in the first for everything except one that we’re in the second [quartile], and the only one we’re below [the second quartile] is colorectal cancer screening.”* **3. needs & Resources of Those Served by the Organization** Barrier: *“There’re challenges. We have, you know, we have language and culture. We have patients from 50 different countries in our clinic.”* *“It seems that people in the community are very, first of all, women are very aware of breast cancer screening, and they come in asking for breast cancer screening…cervical cancer screening is also very well, has been, people know about it and request it. And colon cancer seems to not be as well understood as a condition that needs to be screened.”* *“Most of my patients are adults. And so we have, it’s hard to keep up because I’m generally not talking about colon cancer screening on the first visit. And then, you know, there may or may not be a second visit for a while.”*	**1. Available Resources** Facilitator: *“I think this grant helped to fund our ICS (Integrated Care Specialist), two of our positions…as we grew, we built up and made more of that, those folks.”* Barrier: *“If I have a positive FIT, it’s probably a year before they can get a colonoscopy.”* *“Sometimes things don’t get disseminated as well as we think that they do…we don’t have any, like, easily available for this program specifically.”* *“A lot of stuff ends up into the providers’ hand, so like I have to see, I have to, like, touch everything…which is sometimes good, but mostly just draining.”* *“One of the providers work 80 hours a week. The other one is part-time at 20 hours but puts in probably 60 hours.”* *“The providers here want to do a good job…sometimes you see stuff like that and you go, ‘it’s hopeless’…there are just so many barriers.”* **2. Leadership Engagement** Facilitator: *“The organization as a whole is kind of backing that (CRC screening program) and putting the effort toward it, and putting the time toward it – we had everyone blocked out schedules to get this training.”* **3. Culture** *“Our clinic culture is patient-centered and also we want to work together…it’s very teamwork-orientated.”* *“Everybody is very committed to our mission and doing the right thing and doing things that are going to improve the lives of our patients and people in our community.”* *“Our staff here takes great pride in accomplishing goals and having high achievement levels.”* **4. Organizational Incentives & Rewards** *“Right now, there are no incentives. I think incentives would be nice. I don’t know how much they would help, but I think they would be nice.”* *“I remember getting a gift card because I had the highest screening rate or something at, or like the most FIT. So, to get that recognition that all the work, I’m doing something that all work, I’m doing something good, that’s nice.”* **5. Goals & Feedback** Facilitator: *“Some of our staff were surprised at some of the data…didn’t know that they were as low as they were…that was incorporated into some of that educational, like, slide show that we did with staff…that’s motivating…as we’re trying more to this team-based approach, it helps having everyone on the same page.”* *“The data has helped drive some of these other implementation strategies.* Barrier: *“There have been goals for colon cancer screening kind of globally. I can’t think of what they are offhand. There’s usually a bar that we’re below.”* **6. Tension for Change** *“We want to and need to increase our goals. We understand that we need to increase it.”* **7. Access to Knowledge & Information** “*The grand rounds, we’ve had a few on colon cancer, just basic information. And then on the motivational interviewing…and other stuff, print material. I don’t think there really was all that much print material that was all that helpful.”*	**1. Knowledge & Beliefs about the Innovation** Facilitator: *“I think making sure your patients are healthy…and they’re up to date on these screenings that are advantageous, and encouraging your patients to be an advocate for their own health, or educating patients on this…that’s the incentive for myself to help patients become better advocates of their own health care.”* *“We want our patients to be as healthy as possible, and getting their necessary screenings is always a goal.”* *“We also have a really, really strong team here. Everyone’s really positive, and so I think if we do set a goal and somethings we could, I think that people would be on-board to do that.”* **2. Other Personal Attributes** *“Well, what motivates me would be wanting to do my job well for my patients. I guess a certain amount of self-respect. I’d like to think that I’m a leader amongst providers, so I really do want to see that my numbers are better.”*	**1. Engaging** *“You guys were great…The grand rounds, we’ve had a few on colon cancer, just basic information. And then on the motivational interviewing…and other stuff, print material. I don’t think there really was all that much print material that was all that helpful.”* **2. Executing** *“We have an integrated care specialist, who is doing outreach and identifying people that are likely to need colorectal cancer screening, and them it’s a conversation that comes up.”* **3. Reflecting & Evaluating** *“I think the site-specific rates, provider-specific rates, and the order rates and completion rates, those are all good data.”*
**Integrated Care Specialist (*n*** **= 4)**	**1. Relative Advantage** Facilitator: *“Now they really gave us, like a FIT list that they want us to work from, a FIT list and process. So, we’re working with patients that already given the FIT test. So, we’re just reminding them.”* *“We’re actually following up and we’re really looking into those charts to make sure that patient completed. And we also create care management, and we see that the colonoscopy has been completed, we just put ‘completed’.”* Barrier: *“When we were creating the popup alerts, that was working back then. At the beginning it was working so great because providers would see the notification and they would do the test, the ordering the FIT test. But then eventually, I feel like when they saw that popup alert, like, showing up all the time, they kind of got used to that, so that’s why we decided to change the workflow.”* **2. Adaptability** Facilitator: *“So we didn’t change the workflow. Yes, we kind of, we changed it just a little bit just to add colorectal cancer, because we were doing hepatitis B. And then we started doing both.”*	**1. Needs & Resources of Those Served by the Organization** *“I think it is really…meets the needs because once patients actually do decide to do the screening, and they’re happier if they know they’re healthy.”*	**1. Available Resources** Barrier: *“The only thing that we were using was kind of like leaflet of information from CDC…and information that we were given through the program as well. It was just a pamphlet informing about the different types of screening.”* **2. Culture** *“Our goal is taking care of our patients first…getting them to take charge of their health…for patients to get tested if they need to get tested and treated and we’re always there for them.”* **3. Goals & Feedback** Barrier: *“it would be great to get more communication about where we stand. Because usually, when we hear about our program through our supervisor, it is pretty much to let us know that we didn’t hit a certain number. But we are not aware of what number to hit.”* *“We didn’t have a number set, but we just want to see, to get* better, to get higher. **4. Organizational Incentives & Rewards** *“Nothing…They have not given s anything or said anything to us.”*	**1. Self-efficacy** *“Well, some people say nobody reads what you get in the mail. Yeah, perhaps actually read…so we have all our patients that we’re giving a FIT test, and if they don’t return, we’re calling them, remind them…So a lot of them are actually bringing their FITs back, or they’re calling to schedule an appointment.”* *“Very confident, you know? We do have several places where we check for our patients’ screenings, and we check to make sure they are needed, and we, like I said, we send our flags to the doctor. I feel very confident. I think we’re doing well.”* **2. Other Personal Attributes** Facilitator: *“Well, I speak Spanish, so that sometimes, that helps a lot. I try to really promote, like preventive care visits for our patients, where a lot of that can, that conversation takes place. Because a lot of our patients sometimes don’t understand the need to come in for things when they’re not unwell.”*	**1. Reflecting & Evaluating** “*So we have all our patients that we’re giving FIT test, and if they don’t return, we’re calling them, reminding them…So a lot of them are actually bringing their FITs back or they’re calling to schedule appointment.”*
**Administrator (*n*** **= 2)**	**1. Complexity** Facilitator: *“I think the language in the EMR reminder was straightforward. We knew what it meant – that they were, the patient’s due for colon cancer screening. The provider assessment and feedback, I think it’s, the data is pretty much straightforward.”* **2. Relative Advantage** *“Trying to highlight that and making sure that people are aware of it, as well as talking with the MAs. And you know, it’s like, yes, this orders and alerts and those kinds of things. So I think we’ve done something.”*	**1. Needs & Resources of Those Served by the Organization** Barrier: *“I guess I just think about we have a lot of things that are required…there’s like 15 minutes that each provider has with a patient. And, we’ve got, like, 16 different preventive care kinds of screenings that they need to be aware of.”*	**1. Available Resources** Facilitator: *“We did implement another system that does population health management…that helped us kind of identify patients who are missing colon cancer screening.”* **2. Tension for Change** *“We really have wanted to do, make a more concerted effort and now looking at some different strategies and whatever we can do to improve it (CRC screening rate).”* **3. Goal & Feedback** Facilitator: *“I just started the last two months talking with our CMO, and we also have a new provider that we’ve hired that’s very focused on outcome and quality. And looking at having some dashboards. And having information that’s communicated regularly.”* **4. Networks & Communication** *“We’ve got to push more information out to our providers. And not even just the providers. And that’s where I think the dashboard’s good because it’s going to the team.* **5. Organizational Incentives & Rewards** *“As an FQHC, we really can’t be providing incentives.”*		**1. Reflecting & Evaluating** *“I mean we have increased from like 26, 7% compliance rate to almost like 37, 38% compliance rate over the course of, like, two to three years.”*

### Domain 1: Intervention characteristics

Within the intervention characteristics domain, the codes identified were relative advantage, adaptability, and complexity. The following are themes as barrier or facilitator to implementation efforts:

### Barrier: Perceived burden and provider fatigue with EHR provider reminders

The EHR provider reminder generated alerts when the provider opened the patient’s medical record during visits. Providers needed to respond to the alert by updating the patient’s CRC screening history or ordering a screening test before advancing to the other medical record parts. Although the EHR provider reminder was considered simple and straightforward, some providers grew frustrated and started ignoring the alert knowingly or found ways to bypass them.

### Barrier: EHR provider reminders were not up to date and became unreliable and ineffectual

When patients screen for CRC using the stool-based fecal immunochemical test, lab technicians upload the test results directly to the EHR. However, when patients screen for CRC using colonoscopy, they receive a referral to see an outside provider and complete the colonoscopy at another facility. Most of the time, the colonoscopy results are faxed to the clinic and manually entered into the EHR. Thus, EHR provider reminders might not be accurate or up to date and could create frustration.

### Facilitator: Quarterly provider assessment and feedback reports provided real-time data that motivate changes

Although EHR collects a large amount of detailed patient health information, raw EHR data is disorganized and full of uncodified variables. The quarterly provider assessment and feedback intervention organized raw data from the EHR and provided performance evaluation reports at the provider and clinic levels. Providers could compare their performance with other providers at their clinic, and the clinic could compare its performance with other clinics within the FQHC. The quarterly provider assessment and feedback motivated the desire for changes.

### Facilitator: The implementation of EBIs integrated with workflow processes

The implementation of the EBIs was considered straightforward and integrated into the clinic workflow without significant interruption. The leadership and implementation champions’ support and oversight made the adaptation process run smoothly with less resistance.

### Domain 2: Outer setting

Within the outer setting domain, the codes identified were the needs and resources of those served by the organization, peer pressure, and external policy and incentives. The following are themes as barrier or facilitator to implementation efforts:

### Barrier: Challenges to providing health care services to diverse patient populations

With 82% of patients from racial/ethnic minority groups and 32% of patients speaking a language other than English, it is challenging to provide culturally and linguistically competent care, let alone provide education in CRC screening and persuade them to comply with screening recommendations.

### Barrier: Lack of awareness about CRC screening among patients

Over the years, efforts to promote breast cancer and cervical cancer screening achieved widespread attention, with the national breast cancer screening rate at 78% in 2016 and cervical cancer screening rate at 81% in 2018 [40, 41]. However, the organized efforts to promote CRC screening nationally have just started during the last decade. Furthermore, widespread media promotion might not reach minority communities, especially in communities where members are best served with a language other than English.

### Facilitator: Pressure from funding requirement to report quality measures annually by the Health Resources & Services Administration (HRSA)

All FQHCs must submit data that reflect activities in the HRSA-approved health center project. Furthermore, each year health center grantees must report on their performance using quality measures defined in the Uniform Data System (UDS), such as CRC screening rate. The UDS is a standardized reporting system that provides consistent information about health centers and is open to public access.

### Facilitator: Peer pressure to achieve high performance

UDS currently assigns quartile (1 to 4) to each quality measure. Clinical performance for each quality measure is ranked from quartile 1 (highest 25% of reporting health centers) to quartile 4 (lowest 25% of reporting health centers). Furthermore, in recent years, HRSA has begun providing different Quality Improvement Awards to promote the overall quality, efficiency, and value of the nation’s health centers’ healthcare services. These awards recognize the highest performing health centers and those health centers which have made significant improvements and gains from the previous year. The pressure from competing with other health centers motivates changes and creates an openness to improve the quality of care.

### Domain 3: Inner setting

Within the inner setting domain, the codes identified were implementation climate, readiness for implementation, culture, and network and communication. For the implementation climate, we also identified three sub-codes: tension for change, organizational incentive and rewards, and goals and feedback. Also, there were three sub-codes for readiness for implementation as well. They were leadership engagement, available resources, and access to knowledge and information. The following are themes as barrier or facilitator to implementation efforts:

### Barrier: Absence of CRC screening goals

The target goal for CRC screening rate was not clearly stated, neither at the organizational level nor at the clinic level, which might have hindered the commitment for improvement.

### Barrier: Poor communication on goals and performance

The frontline implementers, such as providers and CRC stewards (integrated care specialists), did not know the organizational goal for the CRC screening rate. The quarterly provider assessment and feedback reports were not communicated directly with CRC stewards (integrated care specialists). They only learned about the performance of their efforts from their supervisors when the numbers were low and had no knowledge about the targeted number.

### Barrier: Absence of print materials for frontline implementers to educate patients

In addition to the patients’ unfamiliarity with CRC screenings, 82% of patients were from racial/ethnic minority groups, and 32% spoke a language other than English. Providers and CRC stewards (integrated care specialists) needed print materials covering various CRC screening-related topics in multiple languages, not just information about CRC and different types of screening methods.

### Facilitator: A culture of teamwork and a patient-centered mentality

The FQHC had a strong organizational culture of teamwork. A team approach is necessary to increase CRC screening since CRC is not a discrete event and involves multiple interfaces with health professionals. Because of the patient-centered mentality, there was a sense of commitment to get patients screened for CRC.

## Discussion

This qualitative study aimed to identify barriers and facilitators to implement three EBIs through stakeholders’ experience in an urban FQHC, determine which areas can be improved, and ultimately provide recommendations for a new project, ACCSIS-Chicago. We identified seven themes under barriers and five themes under facilitators. We identified two facilitators (adaptability and relative advantage) and two design quality and packaging barriers under the intervention characteristics. Our interviewees gave us detailed accounts of how the EBIs fit into their workflow and how the frequent assessment and feedback reports motivated them to change their ordering behaviors and improve CRC screening rates at their clinic. The two barriers we identified were related to the design and packaging of the EHR provider reminder. The provider reminder was a new feature added to the EHR system, and some providers found it frustrating when they could not advance to other features without addressing the prompt. Some providers identified workaround to bypass the prompt; however, such force flexibility may promote burnout [42], which some providers called “prompt fatigue.” Our findings on the importance of various aspects of the EBIs are consistent with studies that have examined how intervention characteristics influence implementation [12, 43, 44].

Prior research has reported how outer setting characteristics, such as patient needs [45], external policies [46], and inter-organization competitive pressure, [47] can influence implementation success. We found that competing with other FQHCs and the reporting requirement from HRSA could facilitate the implementation of EBIs, while a high level of patient needs could hinder the adoption process. Our findings highlight the need for implementing strategies that consider the complexities of the patient population. Within the inner setting, ongoing staff communication has been found to increase the likelihood of EBI sustainability over time [48]. Also, appropriate feedback can benefit EBI implementation and has been associated with higher implementation success [49, 50]. In fact, our study found that poor communication and lack of feedback between leadership and staff could hinder the implementation process, highlighting the benefit of establishing feedback and communication mechanisms. Another notable finding from our study was that a strong culture of teamwork facilitated the implementation process. Studies found that teamwork provides the capacity to solve problems together during EBI uptake [51–53].

The ultimate goal of this study was to identify possible implementation strategies for a new project that can mplement the same EBIs to promote CRC screening in FQHC settings. Table 3 summarizes the actionable areas based on barriers identified and proposed strategies (Table 3).

**Table 3:** Summary of actionable area and propossed strategy

Evidence-based intervention	Actionable area	Proposed strategy
1. EHR provider reminder	• Perceived burden and provider fatigue with EHR provider reminders • EHR provider reminders are not up to date and becomes unreliable and ineffectual	• Use teamwork approach and share the burden ○ Conduct morning huddles ○ Implement standing order for CRC screening ○ Use medical assistant to check, confirm and update CRC screening status during patient intake
2. Provider assessment & feedback (provider- & clinic-level)	• Absence of CRC screening goals at the organizational and clinic levels • Poor communication on goals and performance	• Assist leadership to set realistic goals for CRC screening rate at the organizational level and individual clinic • Include the target CRC screening rates in the quarterly assessment and feedback report • Disseminate the quarterly clinic level assessment and feedback report to all members of the clinic • Provide technical support and financial assistant to create a quality data dashboard within EHR to provide timely feedback
3. CRC stewards (integrated care specialists)	• Challenges to providing health care services to diverse patient populations • Lack of awareness about CRC screening among patients • Absence of print materials for frontline implementers to educate patients	• Identify and collect culturally and linguistically specific CRC education material • Develop CRC related information beyond types of screening methods • Develop a resource guide for frontline implementers

### Proposed implementation strategy for EHR provider reminder

We will tackle the two barriers related to the EHR provider reminder intervention using a teamwork-based approach to reduce the burden of responsibility. The proposed strategies will enhance, not replace, the EHR provider reminder intervention.

#### Strategy 1: Conduct morning huddles

“Huddles” are a structured daily health care team communication process done face-to-face for a brief duration (e.g., 5 to 10 min) and involves a team’s full membership. Huddles provide opportunities for team members to communicate and collectively strategize about managing daily patient demands and workflow, address patients’ unique needs and preferences, and improve the provision of preventive services through previsit planning [54, 55]. The Agency for Healthcare Research and Quality (AHRQ) recommends that healthcare teams huddle every morning for at least 10 min [56]. At FQHCs, huddles can be done in the morning before the clinic begins. During the morning huddle, providers will get updates on patients’ CRC screening status and any perceived barriers from the medical assistant. Making the shift from provider-centric to team-based care can lessen the burden and frustration caused by the EHR provider reminder alone.

#### Strategy 2: Implement standing order for CRC

One strategy to reduce missed opportunities is standing orders. The CDC has recommended standing orders for adult vaccination since 2000 [57]. Standing orders enable nurses and other staff to carry out a medical order according to a practice-approval protocol without a provider’s examination or requirement for approval. Standing orders might empower medical assistants to identify patients who are due for CRC screening and provide them with a home testing kit during a medical visit. Standing orders can free providers to address other health priorities. For standing orders to work, teamwork is essential [58].

#### Strategy 3: Use medical assistants to check, confirm, and update patient screening status during patient intake

Medical assistants can check and confirm the patient’s screening status before the patient visits or during the patient intake, and update the patient’s medical record. They can also follow-up colonoscopy reports and update patients’ medical records accordingly.

### Proposed implementation strategy for provider assessment and feedback

#### Strategy 1: Set realistic goals for CRC screening rate at the organizational and clinic levels

Goals direct attention and action [59]. In organizations, goals give direction to employees about what needs to be done. Specific and challenging goals can lead to better task performance and higher effort, mobilize energy, and increase persistence [60–62]. We will work with leadership to develop specific, realistic, and challenging goals to increase the CRC screening rate at the organizational and clinic levels from the baseline data.

#### Strategy 2: Provide assessment and feedback reports with targeted goals and disseminate them to all staff members

Specific, challenging goals, in conjunction with appropriate feedback, contribute to higher and better task performance [62]. Feedback not only help individuals determine their level of performance, but also determines the adjustments needed to improve. We will include the target organizational and clinic goals for CRC screening rates in our quarterly provider assessment and feedback reports to serve as a performance benchmark and disseminate the report to all staff members.

#### Strategy 3: Provide technical support and financial assistance to create a quality data dashboard within the EHR

One of the strongest facilitators identified during the interviews was the importance of quarterly provider assessment and feedback reports on motivating behavior changes***.*** Although many FQHCs have EHRs, most do not have the capacity to implement EHR generated feedback for clinicians because of the lack of resources and technical support. Although during the study period, the research team provided organized data and feedback, long-term sustainability was lacking. Since measuring and reporting outcome data are essential for health care systems to identify opportunities for improvement [63]; in our new project ACCSIS-Chicago, we will build a more sustainable platform for assessment. Specifically, we will provide technical support and financial assistance for our FQHC partners to create a clinical dashboard that links to their EHR and generates real-time assessment and feedback for their providers, which has been shown to impact the quality of care positively [63].

### Proposed strategy to meet education material needs for diverse patient populations

Health education has always been a vital component of patient-centered care. With the influx of diverse patient populations (e.g., limited English proficiency) into the health care system, the lack of time for patient education during routine visits, the dearth of non-English educational materials, and the high rates of poor health literacy all make the provision of this vital service more challenging to accomplish. For the ACCSIS-Chicago project, the study team will conduct an online search to locate all available CRC and CRC screening-related educational materials, including materials in different languages, and screen the education materials for accuracy and health literacy level. We will also work with frontline implementers to identify education material needs other than the basic information on CRC, such as patient decision aids and a graphic FIT test instruction card that does not require English reading skills. Furthermore, we will develop a CRC patient education resource guide with the patient education materials we developed and found online.

### Strengths and limitations

Our study demonstrates the feasibility of using the CFIR to identify facilitators and barriers across different interventions and capture the dynamics of the implementation context while using familiar implementation science terminology to promote greater transferability of findings. This study also provides the necessary evidence for using the CFIR to conduct a formative evaluation to inform future implementation processes. Furthermore, double-coding transcripts provided a rigorous and consistent application of the CFIR codes. However, the use of template analysis and the application of the CFIR domains and constructs as a coding template might have restricted the identification of non-CFIR-related themes critical to the implementation. Also, interviewees’ recall bias may limit findings since implementing the three EBIs began 2 years prior. Our results represent the experiences of one urban FQHC; therefore, themes identified here may not be transferable to other FQHCs, especially FQHCs operating in rural settings.

## Conclusions

The CFIR comprises five domains and 39 constructs and provides a pragmatic structure to guide formative evaluations and build the implementation knowledge base. Researchers can use the CFIR before, during, and after implementation to identify potential barriers and facilitators from individuals involved in the implementation process. In this study, we conducted a post-implementation formative evaluation using the CFIR to explore what factors influenced the implementation of three EBIs in an urban FQHC. The CFIR, with its clear terminology, allowed us to identify barriers and facilitators to inform future research and provided a template to organize research data and synthesize findings, as demonstrated in this study. Thus, the CFIR has the potential to promote knowledge-building for implementation.

In this study, we identified seven barriers that might hinder the implementation and effectiveness of our EBIs. Our findings were consistent with constructs illustrated in CFIR, supporting its use as a guiding framework. These barriers are common in safety-net settings, such as FQHCs, where daily challenges include diverse patient populations, lack of resources, and competing demands. Provider recommendation is a significant predictor for patient adherence with CRC screening [64–66]. However, the workload and competing demands for providers in FQHCs make provider-centric interventions less effective. A teamwork-based approach using huddles and standing orders to share the burden can overcome some of the barriers facing providers and ensure their engagement and participation during the implementation. Facilitators that are unique for FQHCs, including the requirement from HRSA to submit quality measures, the incentive for improvement, and the providers’ commitment to their patients, all can promote changes and openness to new ways of practice. Together, these drivers of change can mitigate resistance and accelerate the implementation process, ultimately increasing the adoption of EBIs and reducing disparities.

## Data Availability

Not applicable.

## Abbreviations

CRC: Colorectal cancer
FQHC: Federally qualified health center
EBI: Evidence-based intervention
ACCSIS-Chicago: Accelerating Colorectal Cancer Screening and Follow-up through Implementation Science-Chicago
CFIR: Consolidated Framework for Implementation Research
EHR: Electronic Health Record
